# Acute spinal cord injury caused by high-voltage electrical burn requiring a multidisciplinary approach in a resource-limited setting: A case report

**DOI:** 10.1016/j.tcr.2026.101347

**Published:** 2026-04-26

**Authors:** Berhanu Shetie Sefene, Wondwosen Mengist Dereje, Shibabaw Fentahun, Abebe Gelaw, Hailemariam Yohannes Asefa, Andinet Azaje, Nebiyu Bekele

**Affiliations:** a Department of Neurology, School of Medicine, College of Medicine and Health Sciences, University of Gondar, Gondar, Ethiopia; b Department of Surgery, School of Medicine, College of Medicine and Health Sciences, University of Gondar, Gondar, Ethiopia; c Department of Internal Medicine, School of Medicine, College of Medicine and Health Sciences, University of Gondar, Gondar, Ethiopia

**Keywords:** Electrical burn, High voltage, Spinal cord injury, Paraparesis, Case report

## Abstract

**Introduction and importance:**

Tissue damage from an electrical burn could result from either direct electrical current and/or its conversion from electric to thermal energy. Although loss of consciousness, altered mentation, and peripheral neuropathy are the common neurologic sequelae of electrical burns, spinal cord injury can also be the only neurological complication of high-voltage electrical burn injuries. In this case report we present scalp soft tissue defect and acute spinal cord injury following high voltage electrical burn in a 35 years old male patient. We aim to emphasize the importance of multidisciplinary management in improving patient outcomes.

**Case presentation:**

A 35-year-old soldier experienced a high-voltage power live burn injury to the right frontal area of the head. Eight hours after the injury, he developed bilateral lower-extremity weakness and a tingling sensation. Basic investigations like CBC, renal function test, and ECG readings were normal. Both brain CT and spinal MRI studies reveal normal. Concomitant with wound management, physiotherapy was initiated, and he had significant improvement in his weakness by the end of the third week.

**Clinical discussion:**

Despite accounting for less than 4% of burn case, electrical burn have special concerns including the potential for cardiac arrhythmias and compartment syndromes with concurrent rhabdomyolysis. Other types of burns include flame burn and scald burn.

**Conclusion:**

High-voltage electrical burn injuries are serious problems causing variable degrees and types of neurologic impairments. Acute spinal cord injuries are among the less commonly reported problems. Given the limited effective treatment options for neurologic complications, appropriate preventive measures, multidisciplinary management, and long-term neurologic follow-up are important.

## Highlights

• High-voltage electrical burns carry high morbidity and mortality. It also has higher risk of neurological involvement.
• Electrical burns may harm the nervous system via heat, shock, or vessel injury.
• Spinal cord injuries account for less than 6% of neurological problems from electrical burns.
• In resource-limited areas, managing such cases is hard due to lack of facilities.
• Early ICU care and coordinated management of complications improve patient outcomes.

## Abbreviations

BMI: body mass index
CT: computed tomography
CBC: complete blood count
C: cervical vertebrae
ECG: electrocardiography
MRI: magnetic resonant imaging
T: thoracic vertebrae

## Background

Electrical burn injuries are common environmental accidents that could result in injuries ranging from mild superficial skin burns to severe multiple organ dysfunction and death. Tissue damage primarily results from either the direct effect of electrical current and/or its conversion from electric to thermal energy [1]. Neurological complications are not uncommon following electrical burn injuries with around 40–75% of patients with high voltage injuries experiencing some degree of neurological sequelae [2]. Electrical burns to the spinal cord could result in paraparesis, quadriparesis, autonomic dysfunction, sensory disturbance and other sequelae.

## Clinical presentation

A 35-year-old right-handed soldier presented to our hospital one week after sustaining a high-voltage electrical injury from a power line while sitting on top of a public taxi. He suffered burns covering 5% of his body surface area, with an entry wound on the right frontal area of the head and an exit wound on the right gluteal region. At the time of the incident, he experienced a loss of consciousness lasting approximately 2 h. Eight hours after the accident, while attempting to walk, he noticed weakness and numbness in his lower extremities.

He was taken to a primary hospital within 10 h of the trauma, where he was admitted to the ICU. Initial resuscitation included administration of Ringer's lactate. Laboratory investigations showed a white blood cell count of 6.3 × 10^3^/μL with 68% neutrophils, serum creatinine of 0.76 mg/dL, and an ECG demonstrating normal sinus rhythm. During his stay at the primary hospital, wound care was provided twice daily, a high-protein diet was initiated, and physiotherapy was started. His weakness remained stable without progression. He had no prior history of weakness or chronic back pain before the injury.

After one week, he was referred to our tertiary hospital for advanced imaging such as CT and MRI, and for surgical management of his wounds, as the primary hospital lacked these modalities and surgical specialists.

**Fig. 1:**
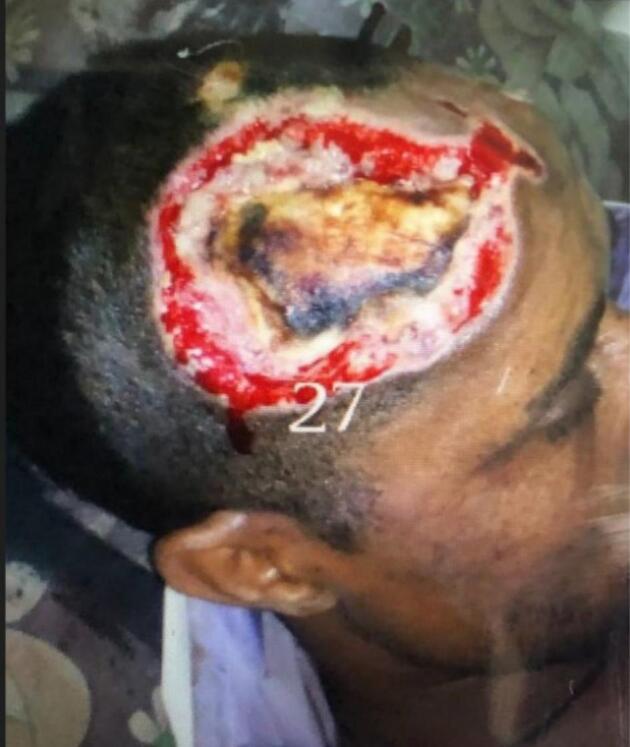
Entry wound on right frontal area.

**Fig. 2:**
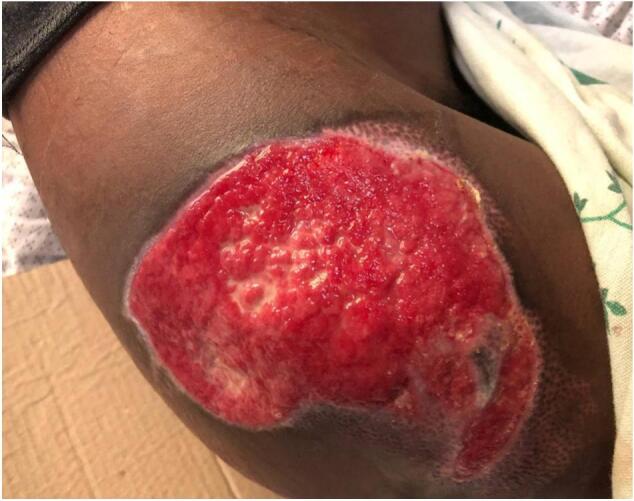
Exit wound on right gluteal area.

Upon presentation to our hospital, the patient was alert and cooperative. Vital signs were within normal limits (BP 100/70 mmHg, pulse rate 80 bpm, respiratory rate 19 breaths per minute, temperature 37.1 °C), and his BMI was 23 kg/m^2^. Physical examination revealed a 4 cm × 5 cm entry wound over the right frontal scalp (Fig. 1) and an 8 cm × 10 cm exit wound over the right lateral gluteal area (Fig. 2).

Neurological examination showed that he was fully conscious and oriented to time, place, and person. Cranial nerve function was intact. Sensory testing revealed loss of vibration and position sense at the level of the great toes bilaterally, while light touch, crude touch, pain, and temperature sensations were preserved. Motor examination demonstrated weakness in the lower extremities, with muscle strength graded 3/5 in flexors and 4/5 in extensors across all muscle groups, accompanied by spastic tone, brisk deep tendon reflexes, and bilateral ankle clonus.

Serum biochemical and hematologic tests were within normal limits. Cerebrospinal fluid analysis was unremarkable, and ECG remained normal. Thoracolumbar MRI performed eight days post-injury revealed a compression fracture of the T9 vertebra and degenerative spondylitis, with the spinal cord appearing normal. A non-contrast brain CT performed in the third week was also normal.

**Fig. 3:**
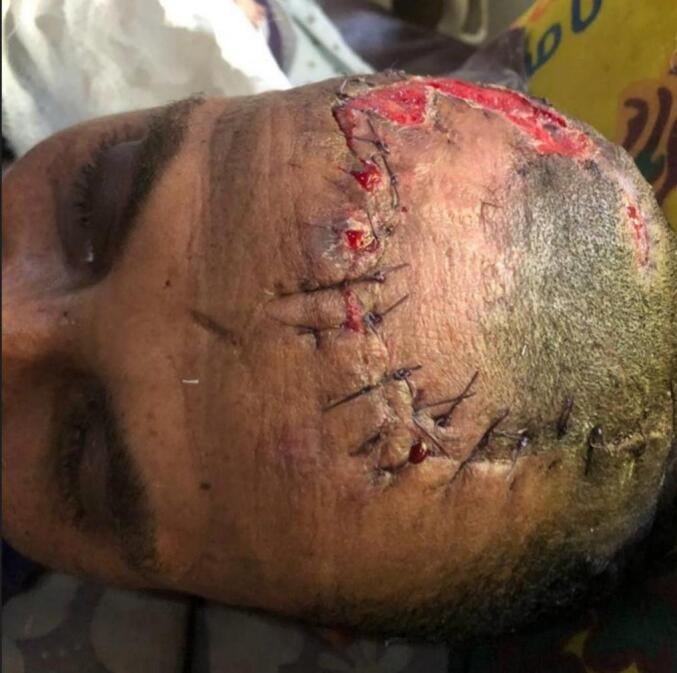
After rotational flap.

The burns were treated with a rotational flap procedure (Fig. 3) and ongoing wound care. Intensive physiotherapy was continued for his weakness. Five weeks after the injury, there was noticeable improvement in lower extremity strength. By the end of the sixth week, he was able to ambulate independently with the aid of a device. He received counseling on nutrition, physiotherapy, and follow-up care before being discharged.

At three months follow-up, he reported no new complaints. Two years after the trauma, he ambulates without any assistive devices, participates fully in normal social activities, and is motivated to return to active military duty.

## Discussion

Spinal cord damage after electrical injury was reported more than 60 years ago [3]. Electrical burns are responsible for as many as 1000 deaths per year in the United States. More than 50% of all electrical burn injuries are nonintentional. While males have seen to be more affected overall, the cause usually being occupational, electrical burns in the household setting are the cause of such injuries among women and children. Individuals in the age group of 18 to 40 years are more frequently affected [4], [5]. The major determinant for the severity of the injury is the voltage of the current that passes through the victim's body, and the nature of the current, that is, direct current (DC) or alternating current (AC). For this reason, electrical burns are broadly classified as follows:

• (I) High-voltage burns: where the voltage is 1000 V or greater. These are more common, accounting for 57.71% of electrical burns. High-voltage burns may be “true high-voltage burns” or there is also an entity called “flash burns,” where an arc of high-voltage current emits heat that causes superficial burns, commonly to the hand and face [4], [6].^,^
• (ii) Low-voltage burns: where the voltage is less than 1000 V, as seen in domestic burns, accounting for 42.29% of electrical burns [4], [6].

High-voltage injuries cause more extensive tissue damage and loss, and may involve more systemic complications like fatal arrhythmias and renal failure or even death, when compared with low-voltage burns. The standard classification of the degree of severity of the burn injury, regardless of the cause, is based on the thickness or depth of the tissue involved in the form of superficial (first degree), partial thickness (second and third degree burns), and full thickness (fourth degree burns) [4].

Nevertheless, it is crucial to emphasize that in electrical burns, the severity of the injury could not be determined based solely on the burned TBSA or on the places of entry and exit of electrical current because, on its course, electricity causes thermal injury in cells and tissues in its path, causing damage to multiple body organs and systems. The ideal time between the occurrence of trauma and the implementation of skin autograft is controversial [7]. High-voltage electrical burns differ clinically from thermal or chemical burns, as they cause much more subdermal damage. Frequently, they cause extensive injury in deeper structures, generating an impact on the central nervous system, consequently leading to the development of multiorgan dysfunction. However, initially, their extent and severity may be underestimated [8].

Apart from the voltage and the nature of the current, it is the duration of exposure and the path taken by the current in the body that determines the extent of the internal injuries, and this is where the conversation can be directed toward the nervous system, which is affected in as much as 21% of cases. The nerves of the body, the large ones being myelinated, and all being natural conductors of the electricity, offer low resistance, similar to blood vessels, when compared with skin, muscle and bone, which offer high resistance. As a result, the nerves and blood vessels favor the path of the current, and this is responsible for the neurological and the cardiac sequelae; while muscles and other soft tissue may suffer the direct impact of the thermal injury generated [4], [6], [9], [10], [11].

Studies have shown that as the electrical current travels through the body, the neurological effects are caused due to the following:

• Electroporation: refers to the opening of pores in the cell membrane, initiating a cascade of events that favor cellular death.
• Vascular injury: due to spasm and release of vascular inflammatory mediators, leading to neuronal ischemia, neuronal chromatolysis, and microglial activation.
• Demyelination: due to alteration of the nature of the proteins and lipids.
• Release of thermal injury, causing destruction of macromolecules.
• Neurohumoral hypothesis: suggesting that electricity aids in circulating substances, specifically cortisol, a stress hormone, and generation of free radicals acting at a distance and by hyper-stimulation of glutamate receptors. The cointeraction of all of these may also be responsible for the delayed effects in the central nervous system (CNS) as well as peripheral nervous system (PNS) [4], [9], [12], [13]. Although rapidly resolving transient spinal cord complaints are common, most patients with spinal cord injuries are left with a permanent disability [14], [15]. Motor deficits occur more often with relative sparing of the sensory component and are secondary to vascular damage incurred by the anterior spinal artery and its branches [16]. This thought is also true in our patient, but what is atypical in our patient is the involvement of the posterior cord as evidenced by affected position sense. The first case of an electrical burn patient with posterior cord involvement was reported by Rodgers A. Fau et al. [17]. In their report, despite the affected position, the MRI was non-revealing by post-injury day 9. But a subsequent MRI of his cervical spine performed on post-injury day 30 demonstrated T2 hyperintensity in the dorsal column of the cervical spine at the C2–3 and C5–6 levels. Similarly, our case has affected position sense, but no feature of posterior cord involvement was detected on an MRI done 16 days post-injury. In patients whose entry sites were the head and neck area Sang Hoon Ko et al. described the associations between the exit site and the pattern of body weakness. Among 13 patients with delayed spinal cord injury, 11 of them developed paraplegia, and the exit sites were lower extremities only, whereas quadriplegia was detected in those whose exit sites were upper extremities [18]. The finding in our patient is also in agreement with this report.

In those with spinal cord involvement, imaging may not show significant changes that explain the body weakness [15]. And in some reports, evidence of spinal cord lesions was detected on repeat imaging days to weeks after the initial one [16], [17]. In our case, both the MRI of the thoracolumbar spinal cord and the CT of the brain did not show significant findings to explain his weakness. The repeat MRI after one month is still normal. Multidisciplinary management involving neurologist, emergency and critical care medicine specialist, neurosurgeon, plastic surgeon, psychiatrist, physiotherapist and ICU nurse professionals improve patient's outcome.

The initial assessment and management of severely burned patients should be similar to the approach of a major trauma patient. However, for the burn patient, the very first step is to immediately stop the burning process and remove burning or hot items from skin contact. Providers should obtain an initial A.M.P.L.E. history (allergies, medications, past medical history, last oral intake, events of injury). The primary survey assesses the A.B.C.s for life threats. In the burn patient, attention should focus on the airway, looking for oral burns that might cause swelling and obstruction, breathing problems from smoke inhalation or lung injury, and bleeding or circulation problems by looking for life-threatening bleeding and checking blood pressure, heart rate, and pulses. The next step would be resuscitation and immediate intervention for life threats. A secondary survey with a complete physical exam follows this [19]. The strategic management of the high-voltage electrical injury can be both challenging and complex. The challenge begins from the time of injury and continues through rehabilitation. The complex aspect of management is the complications that occur due to systemic effects [20]. The management of these severe injuries requires knowledgeable appreciation of the potential widespread anatomic damage and destruction which may be immediately manifest or delayed in appearance. The physiologic responses to the injury, the individual surgical management of the wide variety of wounds, the frequent and varied complications which must be anticipated, and the sequelae peculiar to this type of injury are important determinants in the choice of therapy [21]. The reconstructive plan for electrical burns of the scalp requires consideration of the size, depth, and location of the defect [22], [23]. Management can be challenging with large areas of tissue loss and exposure to the underlying calvarium [23]. The goal is to achieve coverage with well-vascularized tissue while minimizing patient morbidity [24]. Considerations also need to be made regarding the calvarium, as necrotic exposed bone requires the removal of the inner and outer table, if they are not viable, to prevent chronic infection [25]. In our patient the calvarium was viable and rotational flap was done and the patient was discharged after 6 weeks of hospital stay and has significant improvement with no complaint on follow up.

The study has been reported in line with SCARE criteria [26].

## Conclusion

Although high voltage electrical burn as cause of acute spinal cord injury is unusual event when it occurs it often has high morbidity and mortality from cardiac arrhythmias, compartment syndromes and renal insult from concurrent rhabdomyolysis. Closely looking for the above mentioned complications in ICU center is very crucial for patients' survival. Furthermore multidisciplinary management involving neurologist, emergency and critical care medicine specialist, neurosurgeon, plastic surgeon, psychiatrist, physiotherapist and ICU nurse professionals improve patient's outcome.

## CRediT authorship contribution statement

**Berhanu Shetie Sefene:** Conceptualization, Data curation, Formal analysis, Investigation, Supervision, Visualization, Writing – original draft. **Wondwosen Mengist Dereje:** Conceptualization, Data curation, Formal analysis, Validation, Visualization, Writing – original draft, Writing – review & editing. **Shibabaw Fentahun:** Investigation, Writing – original draft, Writing – review & editing. **Abebe Gelaw:** Investigation, Validation, Visualization, Writing – original draft, Writing – review & editing. **Hailemariam Yohannes Asefa:** Validation, Visualization, Writing – original draft, Writing – review & editing. **Andinet Azaje:** Investigation, Writing – original draft, Writing – review & editing. **Nebiyu Bekele:** Investigation, Writing – original draft, Writing – review & editing.

## Consent for publication

Written informed consent was obtained from the patient for the publication of this case report and any accompanying images. A copy of the written consent is available for review by the corresponding author.

## Ethical statement

Ethical approval for this study was provided by the Ethical Committee of our institution.

## Methods

The study has been reported in line with CARE criteria.

## Funding

No funding.

## Declaration of competing interest

The authors declare that there are no known financial conflicts of interest or personal relationships that could have influenced, or appear to have influenced, the work presented in this case report. All authors have contributed independently and objectively, and there are no affiliations or involvements with any organization or entity with a financial or non-financial interest in the subject matter discussed in this report.
